# Development and User-Centered Evaluation of Smart Systems for Loneliness Monitoring in Older Adults: Mixed Methods Study

**DOI:** 10.2196/81027

**Published:** 2026-01-28

**Authors:** Yi Zhou, Jessica Rees, Faith Matcham, Ashay Patel, Michela Antonelli, Anthea Tinker, Sebastien Ourselin, Wei Liu

**Affiliations:** 1 Department of Engineering King's College London London United Kingdom; 2 Department of Global Health and Social Medicine King's College London London United Kingdom; 3 School of Psychology, Faculty of Science, Engineering and Medicine University of Sussex Brighton United Kingdom; 4 School of Biomedical Engineering & Imaging Sciences King's College London London United Kingdom

**Keywords:** older adults, mental health, loneliness, monitoring, user-centered design, smart textile, wearable and ambient technology, wearable technology

## Abstract

**Background:**

Loneliness is a critical issue among older adults and constitutes a significant risk factor for a range of physical and mental health conditions. However, current assessment methods primarily rely on self-report questionnaires and clinical evaluations, which are susceptible to recall bias and social desirability bias, highlighting the need for more objective and continuous assessment approaches. Recent studies have reported associations between physiological and behavioral indicators and the experience of loneliness in older adults. While these technologies have demonstrated correlations between physiological and behavioral sensor data and the experience of loneliness, their implementation has been limited. Most systems rely on fixed-location sensors or smartphone apps, with little attention given to the integration of these tools into users’ daily routines. To date, no published studies have applied smart textile technology, which integrates sensing capabilities directly into garments or furniture, as a medium for loneliness detection. This study addresses that gap by exploring the usability, experiential acceptability, and ethical considerations of smart textile-based monitoring systems.

**Objective:**

This study aims to assess the perceived usability, acceptability, and emotional resonance of a smart loneliness monitoring system integrating sensing garments, furniture, and a mobile app and identify design implications to guide future improvement and promote sustained engagement among older adults.

**Methods:**

Building on earlier conceptual research, a functional prototype system was developed and evaluated through 2 immersive in-person workshops with older adults (N=10). A mixed methods approach was applied, combining structured questionnaires, sensory ethnographic observations, focus group discussions, and experience-based co-design. Quantitative data were analyzed descriptively, and qualitative data were analyzed thematically to explore user perceptions related to system usability, emotional response, lifestyle compatibility, and ethical considerations.

**Results:**

Quantitative data indicated high user satisfaction in dimensions such as comfort, ease of use, and feedback clarity. However, trust in long-term monitoring and willingness to use the system regularly varied. Thematic analysis revealed 4 main areas influencing acceptance, including wearability, usability, and daily integration; trust, privacy, and data control; perceptions of loneliness and the limits of detection; and adoption, applicability, and ethical futures. Participants emphasized the need for discretion, personalization, and human oversight in system feedback and data-sharing mechanisms.

**Conclusions:**

The resulting prototype was positively received, demonstrating the potential of smart systems for passive and personalized loneliness monitoring among older adults. However, adoption is influenced by perceptions of autonomy, emotional sensitivity, and contextual integration. Future development should focus on modularity, transparency, and integration within care infrastructures to ensure ethical and sustainable deployment.

## Introduction

Loneliness and social isolation have been identified as significant global mental health challenges, with particularly profound effects on older adults. There are up to a quarter of older individuals worldwide experiencing social isolation [1-3]. As people age, they may reduce their social interactions due to various factors such as reduced mobility, retirement, or the loss of partners, leading to social isolation and intensify feelings of loneliness [4-6]. A growing body of research has shown that prolonged loneliness is associated with increased risks of depression, cognitive decline, cardiovascular disease, and mortality rates, which poses health risks comparable to smoking and obesity [7,8]. Beyond its impact on individuals, loneliness also places substantial strain on health care systems by increasing demand for clinical care and long-term support services [4].

Despite a growing understanding of the impact of loneliness on health, it remains a challenge to accurately measure and monitor loneliness, particularly in nonclinical and home-based settings [2,7]. While clinical settings may allow for structured assessments by health professionals, such measurements are often constrained by time, context, or the presence of other comorbidities. Traditional assessment methods mainly rely on self-report questionnaires such as the University of California, Los Angeles 3-Item Loneliness Scale, the De Jong Gierveld Loneliness Scale for older adults, or clinician-administered questionnaires [9,10]. While these tools are well-validated, they are susceptible to recall and social desirability bias, particularly among older adults who may underreport emotional distress due to stigma or generational attitudes toward mental health [11,12]. Moreover, such methods often provide snapshot assessments rather than capturing the dynamic, fluctuating nature of loneliness as experienced in daily life [13]. These limitations emphasize the need for continuous, objective, and context-aware detection of loneliness, enabling more timely and personalized interventions.

Sensor-based technologies, especially wearable and ambient sensing systems, have advanced considerably in mental health monitoring, offering new opportunities to detect loneliness through physiological and behavioral data [14,15]. Recent studies have reported several behavioral patterns and physiological indicators associated with loneliness, including reduced physical activity [16,17], sleep disturbances [5], binge or comfort eating [18], elevated blood pressure [19], and increased average salivary cortisol levels [20,21]. While these findings do not establish diagnostic relationships, they suggest measurable correlates that may guide the design of future sensing-based systems. Wearable devices such as smartwatches and fitness bands have demonstrated the ability to monitor many of these indicators. When analyzed over time, these data can provide inferences about an individual’s psychological well-being and deviations from their baseline states [20,22]. Additionally, various sensing systems have been applied to monitor loneliness and social isolation in older adults. These include vision-based motion capture systems for activity level tracking [23], ambient light and sound sensors for detecting social behaviors [24,25], and smartwatches (including accelerometers or inclinometers) to track posture and sedentary behavior [16,26]. However, camera-based systems often raise privacy concerns, and fixture-mounted sensors such as wall-mounted light and sound sensors may lack the portability required for continuous monitoring at home and in community settings [15]. While wearable devices such as smartwatches offer portable sensing, they present their own limitations in older adults, including discomfort with wrist-worn devices, low personalization, and poor integration into daily domestic routines [27,28]. Moreover, many existing monitoring systems focus solely on physical movement or posture and fail to capture the complex emotional and physiological dimensions of loneliness [14,29].

Textile-based sensing technologies, which integrate sensors and conductive materials into fabrics, are able to offer a comfortable and effective solution for long-term mental health monitoring. By integrating sensing capabilities into flexible fabrics, sensing textile systems can passively and continuously collect data without interfering with users’ daily routines or drawing attention to the monitoring process [22,30]. Furthermore, electronic textiles can seamlessly embed into familiar objects such as garments or home furnishings, enhancing both physical comfort and acceptability for older users [31-33]. Despite advances in smart textiles for health monitoring, no textile-based sensing system has been developed specifically for loneliness detection. One prior study explored the use of a textile band to capture speech frequency as an indicator of social interaction, but it did not attempt to evaluate the subjective experience of loneliness or integrate these signals into a mental health monitoring framework [22]. While a growing body of pervasive computing and ambient-assisted living research has focused on detecting loneliness through environmental sensors and wearables [34-36], these systems have largely relied on noncustomizable and device-centric approaches with limited integration into user experiences. In contrast, textile-based systems offer the potential for more seamless, passive, and embodied interaction. However, despite these advantages, they remain underexplored in loneliness-related apps. Previous research has highlighted the critical role of design factors such as format, materials, and sensor placement in user acceptance and sustained engagement with textile-based systems [37,38]. However, few studies have directly integrated co-design, lived experience research, and user-driven evaluation into the development of such systems for older adults. As loneliness is not only just a behavioral state but also a subjective and socially situated experience, the design and development of textile sensing systems need to go beyond engineering efficacy to reflect the emotional, ethical, and contextual needs of users [12]. This study addresses this gap by placing older adults’ voices at the center of system development and evaluation.

In our previous research, we conducted interviews and collected feedback from older users and stakeholders to understand the design requirements and expectations of smart loneliness monitoring systems for older adults [12,27,28]. These earlier works were primarily conceptual, exploring hypothetical interactions and preferences prior to the existence of a working prototype. In contrast, this study advances this body of work by designing and evaluating an integrated smart loneliness monitoring system, comprising sensing garments, furniture, and a companion mobile app through immersive user engagement. Additionally, this study makes a novel contribution by combining prototype-led experience, sensory ethnography, structured quantitative feedback, and co-design outputs to generate both actionable design insights and a deeper understanding of the emotional and ethical responses of older adults. By combining quantitative and qualitative analyses, we examined different dimensions of user acceptance in the context of smart loneliness detection, including wearability, emotional trust, loneliness perception, and pathways to adoption. Finally, we discussed the design implications, ethical considerations, and future directions for integrating smart textiles into the everyday mental health care of older adults.

## Methods

### Overview

This study builds upon our previous interview and co-design research conducted with older adults and stakeholders, which identified essential user needs and expectations regarding the design and development of smart systems to monitor loneliness in later life [12,27,28]. These early insights informed the development of our smart loneliness monitoring systems, which comprise sensing garments and sensing furniture designed to unobtrusively capture physiological and behavioral indicators associated with loneliness. The resulting prototype was evaluated in the current focus group study.

To evaluate and further improve the system, 2 in-person evaluation workshops were held, each involving 5 older adults aged 65 years and older who had experienced loneliness (N=10). The aim of the workshops was to gather experiential feedback and design suggestions from older users. A mixed methods approach was used, integrating sensory observations, self-report questionnaires, focus group discussions, and co-design activities. These methods enabled a comprehensive exploration of users’ practical and emotional feedback to the system, providing pragmatic design insights to guide future development.

### Participants

Participants were recruited using a combination of convenience and purposive sampling strategies, with the aim to engage older adults from diverse life backgrounds and with varying levels of technological adaptability. Recruitment was conducted through 2 main channels. First, the research team directly contacted individuals who had previously expressed interest in the DELONELINESS project [39]. Second, a study invitation was distributed via the PROTECT study newsletter, which reaches over 20,000 older adults across the United Kingdom. For logistical feasibility, only individuals residing within a 50-mile radius of central London were considered from the PROTECT email list. Eligibility criteria included being aged 65 years or older, fluent in spoken English, and having experienced loneliness at some point during their later life (postretirement age). Individuals diagnosed with cognitive impairments or dementia were excluded from participation to ensure that participants could provide informed consent and fully engage with the system interaction and co-design activities. Participants were screened by researchers trained in applying the principles of the Mental Capacity Act (2005) [40], which enabled them to assess an individual’s capacity to participate during the recruitment stage.

Loneliness severity was assessed using the University of California, Los Angeles Loneliness Scale [41], which has been linked to various health outcomes and functional limitations. In total, 10 participants who met the eligibility criteria took part in the study and completed both workshop sessions. This sample size was determined based on recommendations for user-centered qualitative evaluations, which typically involve 3 to 15 participants to obtain experiential insights and design implications in early-stage technology development [42]. Similar sample sizes have been used in published co-design and feasibility studies involving older adults and digital health technologies [43-45]. A total of 10 participants consented and completed both workshop sessions. While small in size, this sample enabled in-depth participatory engagement, iterative feedback, and contextual exploration, which are central goals of this exploratory mixed methods study. Additionally, data collection concluded after 10 participants, as thematic saturation was observed across the 2 workshop sessions, with recurring patterns and consistent feedback emerging during the analysis phase.

### Technology Description

The smart loneliness monitoring systems evaluated in this study consisted of 2 key components, including the sensing garment and sensing furniture. In the previous co-design workshops with older adults and stakeholders, we identified key design factors influencing the older adults’ acceptance of monitoring technologies [28]. Additionally, our prior qualitative research exploring the psychological experience of loneliness in later life informed sensing technology selection and development [12,39]. Building on these insights, our systems were constructed to continuously and noninvasively track physiological and behavioral signals associated with loneliness while remaining compatible with the daily lives and domestic environments of older users.

**Figure 1:**
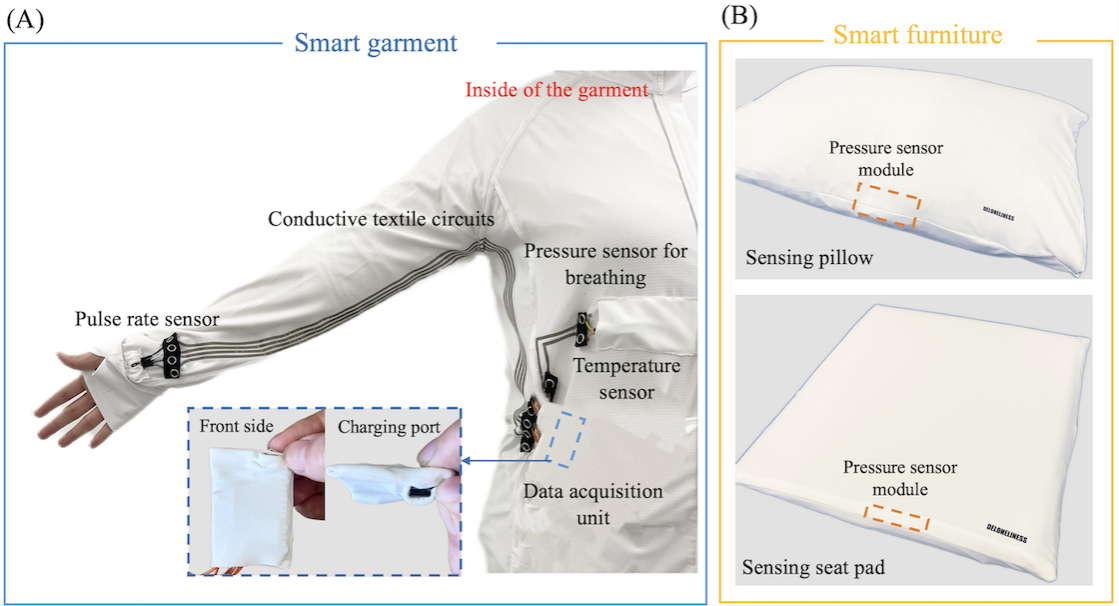
Components of the smart loneliness monitoring system: (A) sensing garment embedded with conductive textile circuits and modular sensors for pulse rate, respiration, and temperature monitoring. (B) Smart furniture including a sensing pillow and seat pad integrated with pressure sensor module for posture and behavioral monitoring.

The sensing garment was mainly intended for physiological signal monitoring. To ensure both wearability and sensing accuracy, we developed 3 different sizes (small, medium, and large) of long-sleeved zip-up shirts made from a breathable elastic textile blend (92% polyester and 8% elastane). The shirts were designed to be worn over regular clothing to facilitate dressing and undressing during workshop sessions. The sensing system of the garment included a fabric-based conductive circuit, modular sensor units, and a data acquisition module. As shown in Figure 1A, textile circuits were mapped along the garment seams and encapsulated using thermoplastic polyurethane via heat-pressing to ensure durability, washability, and smoothness, minimizing tactile discomfort or abrasion risks for older users with sensitive skin.

To support individual autonomy and improve independence, a modular design was applied using metal press-fit snaps, allowing users or caregivers to easily attach, detach, or reposition sensing components without technical expertise. Specifically, a pulse rate sensor was placed at the wrist cuff to enable accurate heart rate monitoring. A temperature sensor and a pressure sensor for respiration were embedded on the interior side of the chest and abdominal regions to collect real-time body temperature and breathing rate data. The data acquisition unit included a built-in inertial measurement unit housed in a soft fabric casing and integrated into a garment pocket. The sensing system was powered by a commercially available rechargeable battery and can be conveniently charged via an external charging port without needing to open the casing, thereby reducing the cognitive and physical burden during maintenance. The proposed modular design allowed older users to customize their configurations by selecting and combining the sensing components most relevant to their individual needs.

The sensing furniture was designed based on everyday household items such as a pillow (sensing pillow) and a seat cushion (sensing seat pad) with custom-developed textile covers (Figure 1B). Pressure sensor modules were embedded into designated internal regions of the pillow and seat pad to enable continuous monitoring of posture and pressure distribution while sitting or lying down. Similar to our sensing garment, the furniture also applied a modular design that allowed sensor modules and the data acquisition unit to be attached via press-fit snaps, simplifying removal for maintenance or cleaning of the textile surfaces.

**Figure 2:**
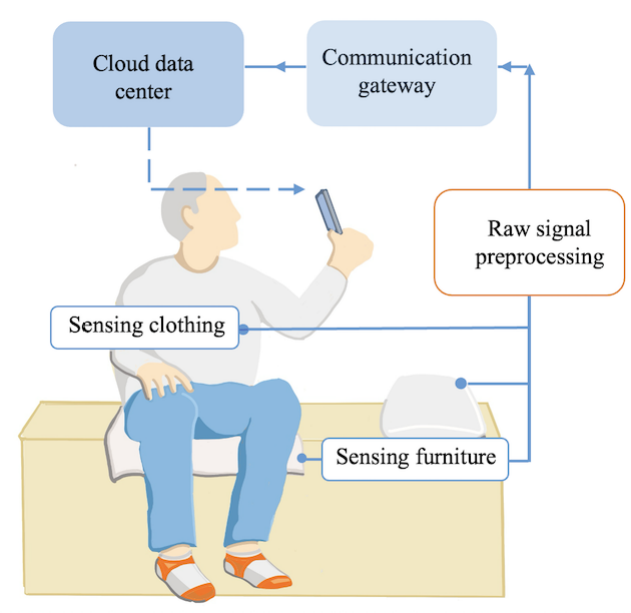
Proposed system architecture and data flow.

Figure 2 demonstrates the system architecture. The proposed smart loneliness monitoring system was designed to continuously collect physiological and behavioral data through the sensing garment and furniture. These raw signals would be transmitted via a smartphone or communication gateway, which performed preliminary signal preprocessing such as noise filtering and timestamping before uploading to a secure cloud environment. In future iterations, advanced machine learning algorithms would be applied in the cloud to identify potential indicators of loneliness, such as irregular activity patterns, reduced physiological variability, or prolonged inactivity. Thresholds for generating loneliness-related feedback have not yet been predefined. Based on participant input during the co-design stage, the system was intended to include adaptive feedback mechanisms in user interfaces, such as customizable mood prompts and check-in features, allowing users to confirm, dismiss, or annotate inferred states.

**Figure 3:**
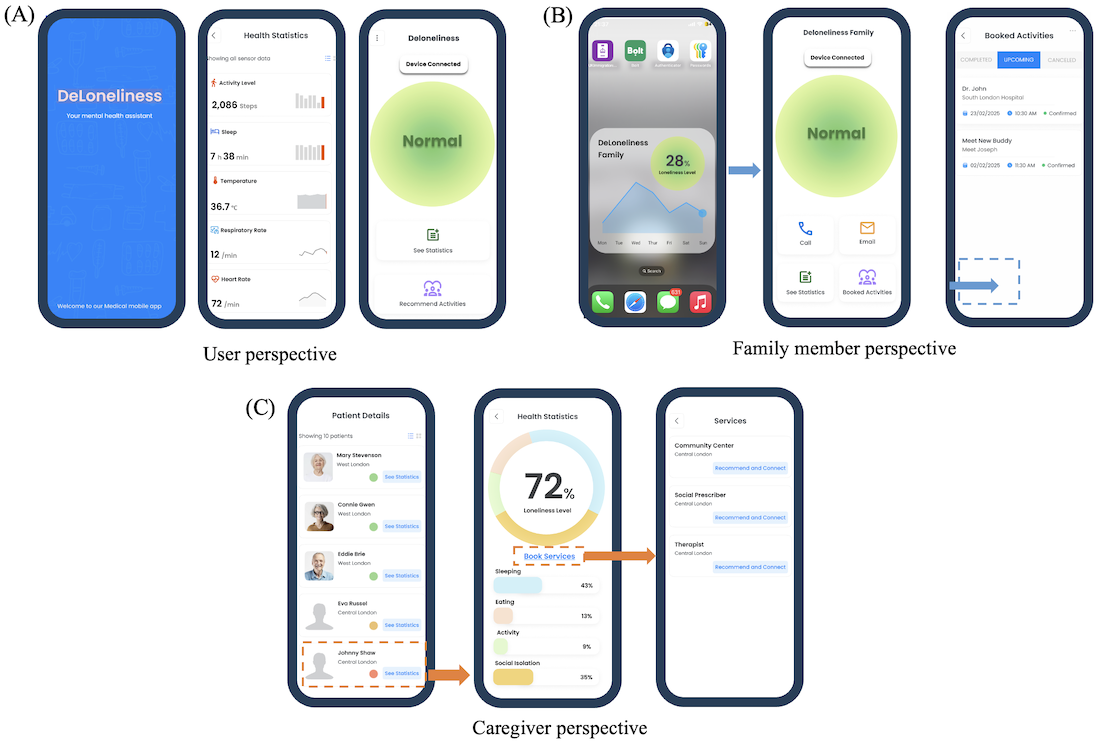
User interfaces of the smart loneliness monitoring system. (A) User interface displaying real-time physiological data, loneliness status, and activity recommendations. (B) Family member interface providing an overview of emotional status, scheduled activities, and communication access. (C) Health care professionals interface showing loneliness metrics across individuals and the service referral interface.

Figure 3 demonstrates our user interfaces from various user perspectives. From the user’s perspective (Figure 3A), the app provided real-time feedback on heart rate, respiration rate, skin temperature, sleep duration, and daily activity levels, while also visualizing current loneliness status and offering recommendations for health-promoting activities. The system further included companion interfaces for family members and caregivers (Figure 3B). Family members can monitor their loved one’s loneliness status, view summarized statistics, and check upcoming social or medical appointments, with integrated communication options such as direct calling or messaging. For health care professionals (Figure 3C), the app offered detailed health data and aggregated loneliness levels across users, facilitating targeted service recommendations based on individual needs.

### Procedure

#### Overview

**Figure 4:**
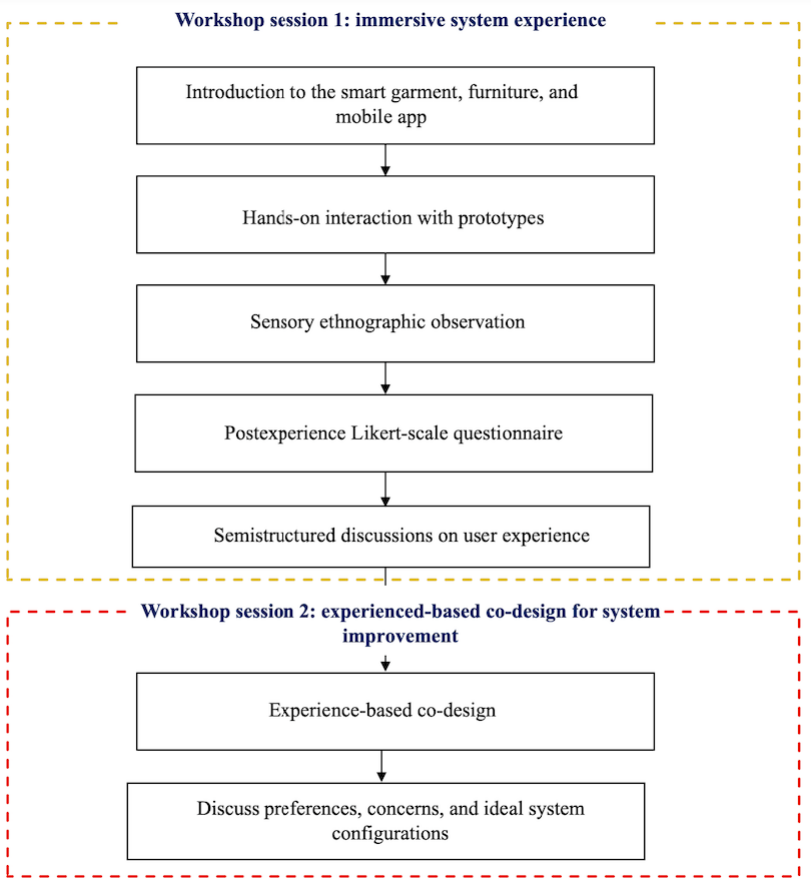
Workshop procedure.

This study was conducted through in-person workshops consisting of a series of structured participatory activities designed to evaluate and improve the smart loneliness monitoring system. The research procedure was divided into 2 sequential components including an experiential evaluation of the system and an experience-based co-design for improvement session (Figure 4).

#### Session 1: Experiencing the Smart Loneliness Monitoring System

The first session of the workshop was designed to allow participants to interact directly with the smart loneliness monitoring system. This session comprised 3 main stages: an introductory demonstration, an immersive trial, and a postexperience feedback phase including a questionnaire and discussion. The session aimed to understand users’ initial perceptions, sensory impressions, and evaluations of the system within a supportive and participatory environment.

The session began with an introduction to the smart loneliness monitoring system by the lead author (YZ), who presented the components of the system, including the sensing garment and sensing furniture. Participants were shown a short video demonstrating the system’s functional workflow and potential use cases in everyday life. This was followed by a live demonstration, during which researchers explained how to operate, maintain, clean, and charge the system. Participants were encouraged to ask questions at any time during the demonstration, ensuring that the purpose, use, and operational aspects of the system were clarified. This stage served as a foundation for the participants’ subsequent independent and immersive system interaction.

All the participants were then invited to try on the sensing garment and interact with the sensing furniture (sitting on the sensing seat pad or lying on the pillow) and operate the accompanying mobile app to see the real-time physiological feedback. This immersive experience was guided by principles of sensory ethnography, which is a commonly applied method in the evaluation of wearable and environmental technologies to capture the embodied and affective dimensions of user experience [46-48]. Participants were encouraged to attend to their physical, emotional, and sensory responses such as how the garment felt on their skin, the sensations caused by the furniture, and their affective responses to the system feedback while experiencing the systems. Three research assistants (YZ, JR, and WL) used structured observation forms (Multimedia Appendix 1) to document participants’ verbal and nonverbal reactions, behaviors, and interactions with the materials.

After the immersive trial, participants completed a Likert-scale questionnaire, designed to assess key dimensions such as ease of use, comfort, perceived usefulness, trust in the system, and concerns about data privacy. The questionnaire was informed by the technology acceptance model (TAM), which is a widely used theoretical framework that explains users’ acceptance of technology based on perceived usefulness and perceived ease of use [49]. Specifically, we adapted core constructs from TAM, including perceived usefulness, ease of use, and behavioral intention to use. To reflect the characteristics of smart textile and ambient sensing systems, we also supplemented items derived from established usability and health technology evaluation frameworks such as comfort, aesthetics, data privacy, and daily life integration [50,51]. A full list of questionnaire items is provided in Multimedia Appendix 2. The experience session concluded with a semistructured focus group discussion. The discussion guide was also informed by the TAM and themes identified in our previous co-design research, which served to ensure comprehensive topic coverage rather than dictate interpretive categories [27]. Topics explored included the system’s integration into daily life, emotional responses to the monitoring experience, clarity and interpretability of feedback, comfort level of the systems, and concerns around data sharing and privacy.

#### Session 2: Experience-Based Co-Design for System Improvement

The second part of the workshop focused on collecting user-driven suggestions for system improvements through an experience-based co-design activity [52]. Based on the insights and impressions gained during the initial system trial, participants were invited by the lead investigator (YZ) to reimagine various aspects of the smart systems. This session aims to empower older adults to become cocreators, allowing them to contribute their experiential knowledge and preferences toward the future development of a more practical, comfortable, and acceptable solution.

Each participant was provided with a design toolkit, which included body and furniture layout co-design template (Multimedia Appendix 3), sensor placement icons, and a range of fabric samples representing commonly used textile compositions (100% cotton, 100% wool, 80% cotton/20% polyester, 80% wool/20% polyester, and 92% polyester/8% elastane). The toolkit also includes materials for conceptualizing sensor integration methods, such as Velcro, magnetic clasps, hooks, and press-fit snaps, which are commonly used to attach electronic modules in textile-based systems.

The session began with a short reflective exercise, in which participants were asked to individually outline their daily routines, experience prompts, and situational preferences related to loneliness and technology use. This laid the foundation for them to anchor design thinking in their own life experience. Participants were then encouraged to engage in open-ended visual prototyping using the templates and materials provided. They annotated body outlines and home layouts with preferred sensor placement zones, marked areas to be avoided for comfort or privacy reasons, and proposed new features such as adjustable garment structures, softer fabric options, or additional sensing functions to enhance comfort, usability, and emotional acceptance.

The co-design session ended with a group sharing activity, where each participant presented their redesigned concept to the group and explained the rationale behind their design decisions. These presentations provided valuable qualitative insights into the different preferences and expectations of older users, which can inform iterative improvements in the future system design.

### Data Analysis

#### Overview

A mixed methods analytical approach was applied to analyze the data collected during the workshops. The study was structured in sequential phases, where the initial quantitative questionnaire was used to prompt structured participant reflection, and the subsequent qualitative phase provided a deeper contextual understanding. Quantitative data from the Likert-scale questionnaire were analyzed descriptively. The qualitative data including audio recordings from focus group discussions, co-design artifacts, field notes from sensory ethnographic observations, and participants’ reflective comments following the co-design session were analyzed using inductive thematic analysis [53,54].

#### Quantitative Data Analysis

Quantitative data from the postexperience Likert-scale questionnaires were entered into SPSS (version 29; IBM Corp) and analyzed using descriptive statistics, including means, SDs, and frequency distributions. These data provided an initial understanding of participants’ perceptions and satisfaction with the system and served as a foundation for subsequent qualitative discussions.

#### Qualitative Data Analysis

Audio recordings from the focus group discussions and the co-design sharing presentations were transcribed collaboratively by the first and second authors (YZ and JR). In addition, co-design artifacts such as annotated body and home layout templates, sticky notes, and sketches were digitized and treated as supplementary qualitative data. Field notes from sensory ethnographic observations were also integrated into the qualitative findings to contextualize participants’ behaviors and embodied responses during interaction with the system.

Thematic analysis followed Braun and Clarke’s [55] 6-phase framework and was independently conducted by 2 researchers (YZ and WL). The researchers first familiarized themselves with the data through review of transcripts and design materials, followed by open coding using NVivo (version 14; Lumivero). Codes were iteratively grouped into broader themes, which were refined through collaborative discussions within the research team. Although the focus group guide was informed by constructs from the TAM and themes identified in prior co-design research, data analysis proceeded entirely inductively. Themes were generated directly from participants’ perspectives and lived experiences, rather than being constrained by a predetermined theoretical framework.

While the earlier quantitative assessment was structured around 8 predefined dimensions, the subsequent qualitative analysis adopted an open-coding approach. This was to ensure that participants’ language, emotional nuance, and contextual expressions were not shaped or limited by prior assumptions. Although some thematic overlap with the questionnaire domains was observed, the 2 phases were intentionally not aligned, allowing for the emergence of novel concerns and experiential insights.

### Ethical Considerations

This study received ethics approval from the King’s College London Research Ethics Committee (reference: LRS/DP-24/25-34602). Prior to participation, all individuals were provided with a participant information sheet and a consent form, which has explained the purpose of the study, their rights, and the voluntary nature of participation. Participants were informed that they could withdraw from the study at any time. Written informed consent was obtained on the day of the workshop. During data processing, all the data were anonymized by assigning each participant a unique and nonidentifiable identification number. A password-protected file containing participants’ names and contact details was stored separately from research data and was only accessible to the core research team. No direct financial compensation was provided for participation. However, refreshments and lunch were offered on the day of the workshop, and travel expenses were reimbursed.

## Results

### Participants’ Characteristics

A total of 10 participants meeting the eligibility criteria took part in the study. Table 1 summarizes their demographic characteristics, including age range, sex, living arrangements, levels of technology use, prior experience with health-monitoring technologies, and loneliness score.

**Table 1:** Characteristics of the participants (N=10).

Characteristics		Values
**Age (years)**		
	Mean (SD)	68.8 (4.2)
	Range	65-79
**Sex, n (%)**		
	Female	8 (80)
	Male	2 (20)
**Highest education level, n (%)**		
	Secondary education or below	6 (60)
	Postsecondary education	4 (40)
**Employment status, n (%)**		
	Retired	9 (90)
	Employed	1 (10)
**Living arrangement, n (%)**		
	Living alone	5 (50)
	Living with others	5 (50)
**Technology use, n (%)**		
	Low	2 (20)
	High	8 (80)
**Experiencing in using health monitoring technology**		
	Yes	5 (50)
	No	5 (50)
**Loneliness score (UCLA^a^)**		
	Mean (SD)	5.1 (1.2)
	Range	3-7

^a^UCLA: University of California, Los Angeles.

### Descriptive Statistics

We conducted a quantitative analysis of the postexperience questionnaires completed by participants following their interaction with the smart loneliness monitoring system. The questionnaire comprised 18 items spanning 7 key dimensions, including ease of use, integration into daily life, perceived usefulness, understanding and clarity of feedback, trust in system functionality, concerns of data privacy, comfort level, and overall system acceptance. Each item was rated on a 5-point Likert scale starting from 1=strongly disagree” to 5=strongly agree.

Table 2 summarizes the descriptive statistics for user perceptions of the smart loneliness monitoring system. Overall, the responses reflected a positive attitude toward the system, with participants tending to “agree” or “strongly agree” with the statements across various dimensions. The highest-rated dimensions were ease of use, understanding and clarity of feedback, and comfort level, with average scores of 4.0 (SD 0.73), 4.5 (SD 0.52), and 4.4 (SD 0.53), respectively. However, responses related to the system’s long-term integration, trust, and data sharing showed greater variability, indicating that evaluations of the system’s role in users’ everyday life were more complex and individualized. These divergences will be further explored in the qualitative findings presented in the Thematic Analysis section.

**Table 2:** Descriptive statistics for user perceptions of the smart loneliness monitoring systems.

Item description			Mean (SD)		FD^a^-1		FD-2		FD-3		FD-4		FD-5
**1. Ease of use**													
	System is easy and convenient to use	3.8 (0.92)		0		1		2		5		2	
	Garment easy to put on or take off	4.4 (0.70)		0		0		1		4		5	
	Maintenance process is manageable	3.9 (0.57)		0		0		2		7		1	
**2. Integration into daily life**													
	Willing to use regularly	3.5 (1.18)		0		2		4		1		3	
	System fits into daily routines	3.7 (0.67)		0		0		4		5		1	
**3. Usefulness**													
	Useful for personal well-being	3.8 (0.82)		0		1		2		6		1	
	Useful for detecting loneliness	3.9 (0.87)		0		0		4		3		3	
**4. Understanding and clarity of feedback**													
	App feedback was easy to understand and interpret	4.5 (0.52)		0		0		0		5		5	
**5. Trust and reliability**													
	Trust in system performance	3.6 (0.69)		0		0		5		4		1	
	System reliably monitors loneliness conditions	3.4 (0.51)		0		0		6		4		0	
**6. Privacy and data concerns**													
	Comfortable sharing data	3.6 (0.84)		0		1		3		5		1	
	Comfortable being continuously monitored	3.8 (1.13)		0		2		1		4		3	
**7. Comfort level**													
	Comfort of sensing garment	4.3 (0.48)		0		0		0		7		3	
	Comfort of sensing furniture	4.9 (0.32)		0		0		0		1		9	
	Comfort of electronic textile component	4.3 (0.48)		0		0		0		7		3	
	Systems feels emotionally supportive	4.1 (0.87)		0		0		3		3		4	
**8. Overall acceptance**													
	Overall system acceptability	3.9 (0.73)		0		0		3		5		2	
	Willingness to recommend to others	3.7 (0.67)		0		0		4		5		1	

^a^FD: frequency distribution.

Specifically, the ease of use received relatively high ratings. The highest-scoring item was “The garment was easy to put on and take off” (mean 4.4, SD 0.70), followed by “The maintenance process is manageable” (mean 3.9, SD 0.57). These results suggest that most of the participants were able to interact with the system independently and comfortably. In contrast, the integration into daily life revealed different perspectives. While most participants agreed that the system could be incorporated into their routine (mean 3.7, SD 0.67), their willingness to use the system regularly over time varied more significantly (mean 3.5, SD 1.18). This points to the complexity of sustained engagement and highlights participants’ desire for personalization and control. These themes are also demonstrated in their co-design for improvement session (see Wearability, Usability, and Daily Integration section). In terms of perceived usefulness, most participants believed that the system would be beneficial to them (mean 3.8, SD 0.82) and could effectively detect indicators of loneliness (mean 3.9, SD 0.87). These ratings support the conceptual value of the system. However, several participants expressed concerns about the transparency of how loneliness was inferred, especially in relation to physiological data. This issue was elaborated further in the focus group discussions (see Trust, Privacy, and Data Control section). Additionally, the dimension of understanding and clarity of feedback was rated highly. All participants reported being able to interpret the outputs provided by the accompanying mobile app (mean 4.5, SD 0.52), indicating a positive perception of the interface’s communicative clarity. However, ratings related to trust and privacy were more mixed. Participants expressed moderate trust in the system’s ability to reliably monitor loneliness (mean 3.4, SD 0.51). Responses to data sharing (mean 3.6, SD 0.84) and continuous monitoring (mean 3.8, SD 1.13) were generally positive, but the higher SDs suggest a divergence in acceptance of long-term monitoring technologies among participants. These differences were explored in depth during focus group discussions, where participants reflected on the roles of caregivers and family members in accessing sensitive data and expressed a wide range of perspectives on appropriate data governance (see the Adoption, Applicability, and Ethical Futures section). Furthermore, the comfort dimension received the most consistently high ratings. Items relating to the comfort of the sensing garment (mean 4.3, SD 0.48), sensing furniture (mean 4.9, SD 0.32), and electronic textile materials (mean 4.3, SD 0.48) showed both high mean scores and low variability, indicating strong consensus among participants. Observational data also support these findings, with most participants exhibiting relaxed body language, positive comments, and tactile exploratory behaviors consistent with physical ease and embodied comfort. Finally, the dimension of overall system acceptance was rated positively (mean 3.9, SD 0.73), and participants reported moderate willingness to recommend the system to others (mean 3.7, SD 0.67). These findings suggest general acceptance and openness to the concept while also indicating further specific design improvement required to support long-term compliance.

Overall, these quantitative results provide an initial understanding of participants’ functional and emotional responses to the system. They also helped shape the focus of the thematic analysis by identifying areas of strong consensus and divergence, which are further explored through focus group discussions, co-design artifacts, and sensory ethnographic observations in the Thematic Analysis section.

### Thematic Analysis

#### Overview

Thematic analysis of the focus group discussions, co-design artifacts, and sensory ethnographic observations resulted in the identification of 4 main themes, each encompassing multiple subthemes, including wearability, usability, and daily integration; trust, privacy, and data control; perceptions of loneliness and the limits of detection; and adoption, applicability, and ethical futures.

#### Wearability, Usability, and Daily Integration

##### Garment Preferences and Adaptive Design

Participants expressed various preferences regarding the design, material, and format of the smart garment. These preferences emphasize the value of adaptable design that can align with individual lifestyles, seasonal changes, and social settings.

One of the most frequently raised concerns was about high temperature during warm weather. While participants acknowledged that the current version of the smart garment featured long sleeves to facilitate wearability within the workshop context, some participants questioned its year-round practicality: “I was just wondering how practical it would be in the summer to wear when it is hot.”

Participants further expressed their expectations for varied design options based on seasonal needs and daily routines:

In the winter, a vest would be something of choice. But in the summer, maybe a t shirt. But then obviously, you’d have to provide different styles.

A couple of participants suggested to design the sensing garment into sleeveless styles such as vests and sports bras, which would allow users to retain their preferred outerwear while still benefiting from the system’s embedded sensing functions:

I would want something that more comfortable and I can put my own clothes on. So for me, I would prefer a vest or something like a sports bra.

Additionally, some older people concerned about the visibility of sensing components, such as metal press-fit snaps and the data acquisition unit. For example, too many metal snaps on the outside of clothing were considered potentially offensive, as participants indicated that they would not want others to know that they were wearing a system designed to monitor loneliness. This reflected broader sensitivities around emotional health and a strong preference for unobtrusive and socially invisible technologies. While some participants found these acceptable on activewear, they felt such elements appeared out of place on more casual garments like t-shirts.

These concerns also extended beyond aesthetic considerations, but also included social signaling and potential stigma. Participants expressed a preference for discreet designs that would not attract unnecessary attention or provoke inquiries:

In a perfect world, I’d like it to be invisible, because otherwise you may spend half your life explaining ... people are going to say “what’s that?” And do you want to discuss the fact that you’re being monitored for loneliness with people you’re not necessarily that close to?

These findings were further supported during the co-design sessions. Participants proposed alternative sensor attachment mechanisms beyond the current snap-on method, suggesting modular sensor units that could integrate with their existing personal clothing. Outcomes from the co-design activities included vest sketches with internal linings to conceal sensor modules and annotations, indicating preferences for “subtle seams” and “concealed fasteners.” One participant also suggested the concept of a “pin-on sensor,” reflecting a desire for wearables that conform to users’ existing dressing habits, rather than imposing new ones. This highlights the importance of designing smart loneliness monitoring systems that seamlessly integrate into users’ daily lives and personal style, thereby enhancing the likelihood of long-term adoption.

##### Sensor Placement and Alternative Technology

During the focus group discussions, participants expressed a range of concerns and preferences regarding the placement of sensors and the physical dimensions of the monitoring components. While most participants were satisfied with the flexibility and comfort of the electronic textile circuit, a key issue identified was the discomfort caused by the rigid data acquisition module within the sensing garment:

About the little hard board (data collection unit), that is to me kind annoying. It’s just wherever you put it.

Some participants also asked whether the hardware could be miniaturized and made less obtrusive, suggesting that improvements in physical design could significantly enhance adoption. This perspective was reflected in several co-design artifacts, where participants reimagined the data module as a smaller patch or accessory. During the co-design sessions, many participants proposed relocating the data unit to the back or side of the garment, thereby reducing bulk and improving comfort around the front torso area.

Beyond physical discomfort, the focus group also revealed diverse preferences regarding sensor location, particularly in relation to wrist-based monitoring. Some participants clearly expressed aversion to wearing anything on their wrist: “I don’t wear anything on my wrist and I don’t particularly want to.”

In contrast, others found wrist-based sensing beneficial, especially those already familiar with commercial wearable devices: “I’m already using my watch to collect my health data, and I find it very convenient.”

These conflicting attitudes were also observed in the sensory ethnographic field notes, which recorded moments of hesitation and hand gestures when participants explored the heart rate sensor embedded within the sleeve of the prototype garment. This further highlights the tactile and cognitive responses that influence user acceptance.

Moreover, several participants raised concerns about the technical requirements for physiological data accuracy. One question reflected a broader concern about the need for reliable biometric sensing: “Do the garment and furniture sensors need to be in close contact with the body to get accurate results?”

This suggests a critical design tension in smart wearable technologies between comfort and data accuracy. Older users preferred sensor systems that offer customizability, modularity, and interchangeable placement. Future iterations of the system should therefore not only miniaturize key components but also offer multiple sensor placement options, enabling users to select configurations that best align with their comfort, personal habits, and lifestyle.

##### Modular Usability

Modularity is a core feature of the system prototype, designed to enhance ease of maintenance, personal adaptability, and user autonomy. Across focus group discussions, co-design outcomes, and sensory ethnographic observations, participants generally endorsed the modular design principle while also highlighting practical challenges related to charging, cleaning, and reassembly.

Some older participants raised concerns about whether individuals with physical limitations would be able to perform these tasks independently:

I didn’t have trouble taking the components out and putting them back, but if one hand wasn’t very agile, it would be hard to do. These steps really require both hands. For anyone older, or with arthritis, it might be difficult to pull these things out of such a small, tight pocket.

This concern was also reflected in co-design artifacts, where participants proposed simplified fastening mechanisms or introduced concepts such as magnetic snap-in connectors to reduce the burden of fine motor control. Ethnographic observations further documented moments of hesitation, uncertainty, or participants seeking assistance when attempting to detach or reattach sensor modules.

Additionally, some participants suggested that the system should include charging notifications within the app:

It’s quite good to know that this stuff doesn’t need to be charged every day like a smartwatch, but should there be a reminder in the app? You know, because you don’t charge it daily, you might forget when it does need charging.

Despite these operational challenges, participants consistently affirmed the value of modularity. They appreciated the separation of electronic components from the textile base not only for practical purposes, such as maintaining and laundering, but also for the potential long-term benefits, such as upgrading or replacing individual components over time. In co-design templates, several participants proposed personalized configurations, suggesting that different sensor modules could be swapped or added according to evolving health needs.

##### Material Comfort and Sensory Feedback

Material comfort is a critical factor influencing participants’ responses to the smart loneliness monitoring system, particularly in relation to fabric texture, thermal regulation, and skin contact. Several participants expressed discomfort with synthetic textiles, especially when worn directly against the skin:

Wearing it as an outer layer is fine, I think it’s quite comfortable. But if I were to wear it close to my skin, like a vest or a t-shirt, I definitely wouldn’t want it to be polyester, because I find polyester too hot and sweaty when worn directly against the skin.

During the co-design activities, participants were given fabric samples, including 100% cotton, cotton blends, wool blends, and polyester-spandex, and they were invited to annotate their preferences directly onto garment outline templates. Cotton and cotton-blend fabrics were the most frequently selected, accompanied by annotations such as “soft,” “breathable,” and “not itchy.”

Comfort was also related to personal experiences and medical histories. For example, one participant noted that clothing design needs to consider changes in tactile sensitivity in the postoperative area:

I had a mastectomy. I don’t have any breasts. I hate anything that scratches. It’s just the normal is not normal anymore. You know what I mean?

Compared to the sensing garments, the sensing furniture components such as the sensing pillow and cushion were generally perceived as more comfortable and less intrusive. This distinction was further supported by sensory ethnographic observations, which captured some participants’ nuanced physical interactions with the garments. While wearing the smart clothing, some participants often made subtle adjustments to collars, pulled at sleeves, or ran their fingers along seam lines. Some participants hesitated before putting on the garment or asked whether the sensors would touch their skin directly. In contrast, interactions with the smart furniture were more relaxed. Most participants sat down without instruction, leaned back comfortably, and engaged in conversation while using the seat pad.

##### Contextual Integration and Lifestyle Compatibility

Participant feedback indicated that the acceptability of the system was closely related to its ability to seamlessly integrate into users’ everyday lives, domestic environments, and personal routines. For example, smart furniture components were generally perceived as less intrusive and more acceptable for long-term and low-effort engagement:

People do tend to sit in the same seat every day, in the same place to watch TV. If someone was sitting there all day watching TV or doing something else, you’d find it very useful.

In contrast, concerns were raised about the disruption that smart garments might cause to the unpredictability of daily routines. One participant described how the demand for continuous wear might not be compatible with their lifestyle:

I do so many things during the day. When I come back from the garden, I might have sweated or gotten dirty and need to change clothes and then, oops, I might forget to put on the smart garment again.

This issue was further reflected in sensory ethnographic field notes, which captured several participants expressing uncertainty about whether they were “wearing it correctly” or whether the sensors would still function properly after shifting position.

During the co-design sessions, participants engaged with home layout templates, marking preferred sensor locations. They often placed sensors in areas associated with habitual furniture use, such as a reading chair, dining table, or frequently used seating areas. Some participants also proposed to integrate the system as part of standard domestic infrastructures in care homes: “You just install it when someone moves in for safety.”

These insights suggest that smart loneliness monitoring systems should not only be conceived as stand-alone technologies, but rather as components within a broader ecosystem of smart living, with potential to be embedded into existing domestic practices and infrastructural frameworks.

#### Trust, Privacy, and Data Control

##### Conditional Trust and System Reliability

Participants across both workshops expressed a degree of trust in the smart loneliness monitoring system but consistently emphasized that trust in such technologies is not taken for granted. Several participants highlighted that trust would need to be earned over time, through demonstrable functionality and accuracy in real-life use:

I think I’d have to actually wear it and see it identify (my loneliness) without me saying anything. That would be the only way to really learn that it was working. What would happen at the end of the day? Would it give you a ping? Something to say “woo! Looks like you’re lonely at the moment.” And if I was in the middle of having a conversation with somebody and feeling perfectly fine, I know I would not trust it.

Moreover, past experiences with commercial wearable monitoring devices appeared to shape users’ current skepticism. One participant referenced their partner’s experience:

My husband uses a Fitbit, but it clearly doesn’t record his steps accurately. So I can’t fully trust it.

These encounters with inaccurate sensing may contribute to reserved user fatigue or doubts about the credibility of wearable sensors. These doubts also extended to smart monitoring systems, especially when applied to emotionally complex and subjective states like loneliness.

While most participants felt confident interpreting the outputs presented in the accompanying mobile app, they nonetheless highlighted a need for greater transparency and interpretability in system feedback. During the co-design sessions, participants proposed a range of suggestions to improve algorithmic explainability, including the addition of visual indicators such as:

Why is it saying I’m lonely?

What data triggered this message?

Another recommendation was the inclusion of a feedback confirmation mechanism, enabling users to validate the system feedback, thus contributing to a dynamic and trust-building model. One participant proposed a “check-in” feature, whereby if the system identified them as potentially lonely, they could choose to confirm or dismiss the notification. Over time, such feedback loops would allow the algorithm to learn from user responses, thereby refining its accuracy and building user confidence.

##### Customizable Data-Sharing Preferences

While participants acknowledged the potential value of sharing data with others particularly in the context of health or emotional support, several older adults highlighted the importance of retaining control over their data-sharing choices:

I feel that collecting this data all the time is quite intrusive. I might not want my daughter to know how I’m feeling at that moment, and I certainly don’t want her using an app to monitor me. I want to stay in control of my emotions, and I feel this would take that control away.

Conversely, another participant expressed openness to sibling-based support, provided that geographic distance warranted it. Interestingly, when asked, “You wouldn’t want your daughter to see your data, would you want to see your mother’s?” the participant hesitated. These responses highlight how data sharing preferences are relational and context-dependent and may vary depending on the role of the recipient as caregiver or care recipient.

Additionally, several participants expressed a preference for conditional sharing models, where data would only be shared under specific circumstances:

If I’m having a breakdown at home because I’m feeling lonely and haven’t seen anyone for three weeks, then I do want them to know. But if I’m just feeling a bit off and can’t be bothered to go to the coffee shop, I don’t necessarily want to share that.

During the co-design sessions, participants proposed improvements to the current permissions interface. Users expressed a desire to select which types of data such as emotional states, activity levels, or physiological indicators would be visible to specific recipients, including family members, clinicians, or community caregivers. Some also suggested adding a dashboard that clearly shows “who can see what,” along with visual indicators to support transparency and ease of management.

##### Ethical Concerns and Data Ownership

Across both workshops, while the majority of participants expressed openness toward the use of monitoring technologies, they also emphasized that trust in such systems depends not only on accuracy but also on transparency of purpose, data flow, and long-term governance.

A recurring concern was “Who owns the data?” and often followed by anxiety about potential commercial exploitation. As one participant asked: “What if the data gets sold? Who’s to say it won’t be sold?”

The prospect that personal health or emotional data might be commodified was troubling for many older adults, particularly given the lack of clear regulation surrounding data collected outside clinical systems.

Concerns also extended to the broader implications of artificial intelligence governance in connection with wearable monitoring systems: “I understand that these technologies are developed with good intentions, but I do worry that they could be repurposed for surveillance or behavioral manipulation.”

During the co-design sessions, many older adults indicated that these ethical concerns did not necessarily result in rejection of the system. Rather, they expressed a desire for greater clarity regarding the system’s governance model, data stewardship, and effective plans for future use. These findings suggest that ethical acceptability is not solely a matter of obtaining “informed consent” at the point of use but requires ongoing transparency and participatory data governance. Future iterations of the system should consider the development of interactive tools that clearly and accessibly communicate key information regarding data provenance ownership and rights.

#### Perceptions of Loneliness and the Limits of Detection

##### The Subjectivity of Loneliness

Some participants believed that loneliness is fundamentally a subjective and emotionally complex experience, one that cannot be directly inferred from behavioral or physiological signals alone. This perspective occurred in our earlier focus group discussions, where several participants questioned the assumption that sensor data could reliably infer emotional states: “What you feel inside can’t be monitored by anyone, no sensor can pick that up.”

Participants also noted that loneliness is highly individualized and not necessarily linked to physical solitude: “You can feel lonely in a crowded room but feel fine when you’re alone.”

This further raised questions among participants about whether the algorithm could accurately identify individualized feelings of loneliness. In response, the investigator (JR) clarified that while current algorithmic models may be developed from broad datasets, their core functionality is intended to adapt to individual patterns. The system is designed to learn over time with correct or incorrect feedback, establishing and refining a personalized emotional profile, thereby improving its accuracy.

Moreover, participants noted that loneliness is not exclusive to older adults but occurs across different ages and life stages, highlighting the need for the system to detect emotional nuance rather than demographic generalization.

In the co-design sessions, some participants expressed discomfort with the term “loneliness,” describing it as “too strong” or “too negative.” They proposed softer alternatives, such as “reflective state” or “well-being indicator.” Others annotated their interface sketches with prompts like “How are you feeling today?” in place of system-generated loneliness labels. These design annotations suggest that users may prefer tools that prompt self-reflection, rather than systems that presume to define their emotional states on their behalf.

##### Algorithmic Assumptions and Multimodal Analysis

As discussed in the previous subsection, participants questioned the logic behind the algorithm used for loneliness detection. For instance, they expressed concern that feedback based solely on low physical activity might lead to false positives: “You’re detected as being very still, but you’re not lonely, you’re just enjoying your book.”

In response, researchers explained that the current system applies multimodal integration, combining physiological signals with behavioral data for a more comprehensive analysis. While participants appreciated this approach, they also asked whether the system could incorporate additional personalized factors to further improve detection accuracy: “If I’m motionless, and those factors you mentioned lead the system to assume I’m lonely, then I’m wondering, what other factors could be added?”

This desire for individualized calibration was also evident in the co-design artifacts. On the smart garment and furniture templates, some participants annotated notes such as “Don’t assume lack of movement means I’m feeling low.” Others, particularly older adults, proposed that the system should seek user confirmation before tagging as a loneliness event, effectively embedding emotional subjectivity into the algorithmic process.

#### Adoption, Applicability, and Ethical Futures

##### Adoption Among the Older Adults

While participants generally recognized the value of the systems in their later life, they also noted that those who might benefit most should be very old adults or individuals strongly invested in their independence (may be the least willing to adopt it). Factors such as psychological resistance and identity-related concerns were seen as major potential barriers to real-world implementation.

Most participants in both of the workshops were still socially active and engaged but reflected on family members who were socially isolated yet unwilling to engage with monitoring technologies or accept support:

My mum is very old and lives on her own. She’s obviously lonely, but there’s no way she’d get involved in something like this.

Others expressed similar insight:

She won’t accept any help. Yes, even though she’s clearly spiraling because of it.

These reflections highlight a tension between need, self-perception, and autonomy. The concept of “independent living” was both a source of pride and a practical barrier. Older adults may perceive acknowledging loneliness or using assistive technologies as a threat to their autonomy or even as an admission of vulnerability.

This resistance was also observed in the sensory ethnographic field notes. While most participants interacted with system components during the experience, a small number showed hesitation or disengagement when asked to wear the garment or respond to app prompts. Some made dismissive remarks such as: “This seems something for people who need looking after.”

In co-design sessions, some participants openly stated that they found it a bit difficult to imagine themselves using such a system even hypothetically because it felt “irrelevant” or “only for people in worse situations.” These views may reflect a desire to maintain a sense of competence and independence, even in the face of known risks or increasing social withdrawal.

##### Timing of App

During the focus group discussions, participants also reflected on when these technologies should be applied. Some participants thought it would be best to introduce the system before severe loneliness or functional decline occurs: “Maybe you need to catch people before they get to that point, so you can do it as a prevention rather than a late intervention.”

Some participants thought that if people fall into severe isolation, their willingness to engage with new technologies may be significantly reduced: “By the time they need it, they may not want to learn it.”

In addition, participants also mentioned that they may be more willing to use the system when they still feel in control, an insight that is consistent with the discussion in the Trust, Privacy, and Data Control section that trust is built through gradual voluntary participation rather than sudden or mandatory use.

Some participants proposed a “onboarding stage” during the co-design session, such as “Let me start with one feature first” or “Phase 1: Activity tracking only, no reminders yet.” Others suggested setting up a “trial period” for the system so that users can experience it during this period without worrying about data being misunderstood or shared too early.

##### Expanded Health Monitoring

While loneliness detection was the primary aim of the system, many participants expressed strong interest in expanding its functionality to support broader physiological and behavior monitoring: “It’s good to know the system can monitor so many different things, but could it also include more features?”

Some participants also highlighted their specific personal monitoring needs: “I often need to drink water, otherwise I get heart palpitations. could it tell me if I’m dehydrated?”

These ideas were further developed during the co-design activities, where participants added additional monitoring points to the smart garment framework diagrams. Suggestions included integrating electrocardiography and blood pressure tracking and hydration-level detection. Rather than replacing the core functionality related to loneliness, these suggestions were seen as complementary integrations that could increase the system’s everyday utility and relevance by addressing users’ broader well-being in a more holistic and meaningful way.

##### Linked Intervention

When participants reflected on the system’s potential to monitor physiological and behavioral signals, the discussion naturally extended to scenarios involving health emergencies and critical incidents. Many raised concerns about automated alerts and connected interventions:

If you’re in a state of severe loneliness or at some kind of risk, the next level of concern is whether social services would actually respond, whether your GP would be notified, or a nurse, or a district nurse would come out. That’s the real worry.

While many participants appreciated the system’s potential to issue alerts or prompts, they expressed hesitancy about fully autonomous system actions, particularly in the context of emotional monitoring. Some expressed discomfort with the idea of automated triage, instead showing a preference for human-mediated intervention: “I would like a caregiver or clinician to review the data before contacting me.”

In the co-design sessions, participants proposed customizable alert settings, including adjustable emergency thresholds, such as “Only trigger an alert if an abnormal signal persists for more than 10 minutes.” and “Notify family members first, then professionals.” These suggestions reflect a strong desire for tiered intervention logic, whereby users can define the severity of signals required to activate alerts as well as the order and type of recipients to be notified. This approach highlights the need for a personalized response, rather than a one-size-fits-all automation model.

Furthermore, although stakeholders such as care providers were not present in either workshop, participants actively imagined various relational configurations. Some participants preferred family members as first contacts, while others, particularly those living alone, favored designated professional care networks.

## Discussion

### Principal Findings

This study examined the acceptability and perceived usability of a novel smart loneliness monitoring system for older adults, comprising sensing garments, furniture, and a companion mobile app. Through user-centered evaluation and experience-based co-design, this study aimed to comprehensively explore users’ practical, emotional, and ethical concerns to the system and to provide actionable design insights for future development. While prior studies in pervasive computing and ambient-assisted living have focused on detecting behavioral correlates of social isolation, few have investigated how older adults themselves experience, interpret, and negotiate such monitoring systems [34-36]. Therefore, our findings contributed to bridging the gap between technical feasibility and user acceptability in the emerging field of smart mental health textiles.

Previous research has shown that older adults’ acceptance of smart monitoring systems is heavily affected by perceived usefulness, particularly whether the data collected support meaningful or supportive interventions [17,56,57]. Given the practical and societal significance of loneliness monitoring, our prior research had explored older adults’ initial design needs alongside stakeholder perspectives at the conceptual level [27]. Building on this foundation, this study combines smart textile design and sensing technology to develop prototypes that allow participants to physically experience, evaluate, and reimagine the system. This study identified 4 main domains affecting user acceptance and future design improvement, including wearability, usability and integration; trust, privacy and data control; limitations of loneliness monitoring; and future adoption, applicability, and ethical considerations.

Our findings highlighted the importance of adaptability and lifestyle compatibility in determining system acceptability, which are key aspects emphasized in previous research on wearable health technologies for older adults. Prior studies have shown that comfort, convenience, and discretion strongly influence engagement with wearable devices, particularly among older users who may have heightened sensitivities to fabric texture or skin contact [15]. In our study, although most participants were satisfied with synthetic materials for outerwear use, individual preferences and health history highlighted the value of personalized textile options. Participants appreciated the modular design and comfortable electronic textile integration, which enhanced wearability. However, unlike previous work that primarily assessed ergonomics or fit, our findings underscored the importance of emotional comfort and social invisibility. Participants expressed concerns about the visibility of sensing components and the potential stigma associated with being perceived as “monitored.” This highlights an important extension of the current understanding of usability from physical comfort to psychosocial comfort. Additionally, the discussion around seasonal practicality and clothing preferences, such as the suggestion to adopt undergarment formats like vests or sports bras, introduces a novel consideration for thermal and social appropriateness and compatibility with individual everyday routines. While some previous smart garment research explored aesthetic design [58], our findings suggest that adaptability to seasonal routines and existing clothing habits is critical for long-term adoption. Moreover, the discomfort caused by rigid sensor modules further emphasizes the need for miniaturized and flexible electronics, a challenge also noted in emerging literature on e-textile scalability and integration [59,60]. Compared to sensing garments, sensing furniture was perceived as more comfortable, less obtrusive, and better aligned with habitual behaviors.

Trust, privacy, and data control were also key to user acceptance. Prior studies on digital health and remote monitoring technologies have consistently shown that trust and perceived data security are prerequisites for sustained engagement among older adults [61]. Our findings support these observations but extend them by revealing that participants’ conditional trust depended not only on privacy assurances but also on the reliability, interpretability, and transparency of system feedback. These aspects highlighted in this study collaborated with previous studies on smart home and telehealth systems. However, unlike earlier work that primarily emphasized the role of institutional trust in health care providers [62], our participants focused on personal data sovereignty, and they want to see, understand, and adjust what the system infers about them in real time. While many older adults found the app’s real-time feedback intuitive and easy to use, their past experiences with commercial wearables, such as smartwatches and fitness trackers, triggered skepticism regarding its accuracy when interpreting subtle emotional or behavioral changes. This echoes concerns in prior research that algorithmic opacity undermines user confidence in affective or well-being monitoring [63]. Our findings show that trust must be earned gradually through use and supported by transparent feedback mechanisms that allow users to confirm or challenge system outputs, which is an important element rarely addressed and discussed in earlier studies. Users have also shown a great preference to granular control over data sharing, allowing users to tailor access to different stakeholders such as family members, clinicians, or caregivers. While prior work on privacy in older adults has discussed consent management in general terms [64], our participants preferred a dynamic and contextual control that could match their current mental state and relationships. These insights extend the current literature by emphasizing that ethical acceptability is not achieved solely through initial consent, but through ongoing transparency, accountability, and user agency in data governance. Future development of wearable monitoring technologies should move beyond data protection compliance to include user-facing transparency features and active participatory data management frameworks.

### Strengths and Limitations

The key strengths of this study lie in its multimethod integration of quantitative and qualitative approaches, combining structured questionnaires, focus groups, sensory ethnography, and experience-based co-design. This enabled a multifaceted understanding of older adults’ experiences with the smart loneliness monitoring system and helped identify comprehensive user-driven directions for iterative improvement. However, several limitations also need to be acknowledged. First, all the participants were recruited from the United Kingdom, which may introduce a degree of regional bias and limit the generalizability of the findings to older adults in other geographic, cultural, or health care settings. In addition, the sample included relatively few male participants. Given that sex may influence perspectives on technology, privacy, and well-being, future research should aim to increase sex diversity to improve the representativeness of findings. Participants with diagnosed cognitive impairment were excluded during recruitment. Researchers applied the principles of the Mental Capacity Act (2005) to assess participants’ capacity to understand the study and provide informed consent [40]. While this ensured ethical participation and meaningful engagement with the system, the study does not include the perspectives of older adults living with cognitive impairment or dementia. Future work should explore how smart loneliness monitoring systems can be adapted or tailored for these populations, who may have different usability needs and vulnerabilities. Additionally, although participants met the inclusion criteria of being aged 65 years and older and having experienced loneliness, the majority of participants remained socially active, potentially limiting the generalizability of findings to more isolated or vulnerable populations. Moreover, the overall sample size was small. While small-scale qualitative studies can offer rich insight, findings should be interpreted as exploratory and hypothesis-generating rather than definitive. Finally, while the workshops were conducted in a controlled environment to allow in-depth interaction and observation, this setting may not fully capture the complexity of real-world use. To further strengthen the validity and practical relevance of the system, future research should involve longitudinal field testing in home or community settings. This would allow for continuous data collection over an extended period, enabling more robust analysis of behavioral and physiological patterns, as well as user engagement over time.

### Conclusions

This study examined older adults’ experiences with smart loneliness monitoring systems, including sensing garments, furniture, and a companion mobile app. Through immersive workshops combining structured questionnaires, focus groups, sensory ethnographic observation, and experience-based co-design, we investigated older users’ practical, emotional, and ethical responses to the system, providing actionable design insights to inform future development. These findings indicate that while participants generally accepted the concept of monitoring via smart textile wearables and furniture, their willingness to adopt such systems over time is highly dependent on usability, personalization, lifestyle compatibility, and perceived control. Participants consistently emphasized the importance of adaptable design that respects bodily comfort, domestic routines, and personal identity. Moreover, the modular smart system developed in this study demonstrates strong potential as a discreet and passive sensing platform for psychological and social health indicators. However, continued user-centered iteration, broader real community testing, and deeper integration with health care infrastructure are required to ensure its future success in real-world applications.

## Appendix

Multimedia Appendix 1 Structured observation form.

Multimedia Appendix 2 Likert-scale questionnaire.

Multimedia Appendix 3 Co-design template.

## Abbreviations

TAM: technology acceptance model

## Data Availability

The datasets generated or analyzed during this study are available from the corresponding author upon reasonable request.
