# Supplementary material for: Development and User-Centered Evaluation of Smart Systems for Loneliness Monitoring in Older Adults: Mixed Methods Study
Source: J Med Internet Res. 2026 Jan 28;28:e81027. doi: 10.2196/81027 (PMC12895156; doi:10.2196/81027)
Supplement: Multimedia Appendix 1 [file jmir_v28i1e81027_app1.pdf]

Evaluation of Smart Textile Systems for Monitoring Loneliness in Older Adults

Observation Form

Participant ID/ Time	Experience Content	Behavioral Observations	Non-Verbal Responses	Quotes	Notes – Feedback Direction
P01- 10:30AM Example	*Garment / Furniture (seat cushion, bed sheet)- Example*	*Adjusting clothing position or tightness/ Repeatedly touching the fabric/ Prolonged screen gaze or gaze avoidance- Example*	*Facial expressions/ Hand gestures/ Changes in posture- Example*	*"The garment feels a bit warm." "I'm not sure what it's measuring." …… Example*	*Comfort level; Acceptance level; Usability; Emotional response; Concerns; Privacy, etc. Example*
