# Supplementary material for: Development and User-Centered Evaluation of Smart Systems for Loneliness Monitoring in Older Adults: Mixed Methods Study
Source: J Med Internet Res. 2026 Jan 28;28:e81027. doi: 10.2196/81027 (PMC12895156; doi:10.2196/81027)
Supplement: Multimedia Appendix 2 [file jmir_v28i1e81027_app2.pdf]

Rating Questionnaire - **Experience the Smart Textile Systems**

Name:

1. After experiencing the systems and use process, to which extent do you agree or disagree with the following statements:

1.1. I find the system easy and convenient for daily use.

1	2	3	4	5
Strongly Disagree	Disagree	Neutral	Agree	Strongly Agree

Please explain your reasons if you disagree: (Optional)

1.2. The garment was easy to put on and take off by myself.

1	2	3	4	5
Strongly Disagree	Disagree	Neutral	Agree	Strongly Agree

Please explain your reasons if you disagree: (Optional)

1.3.The maintenance required for the garment and furniture (such as washing and charging) seems practical for daily life.

1	2	3	4	5
Strongly Disagree	Disagree	Neutral	Agree	Strongly Agree

Please explain your reasons if you disagree: (Optional)

1.4. I would be willing to use this system regularly over a long period of time.

1	2	3	4	5
Strongly Disagree	Disagree	Neutral	Agree	Strongly Agree

Please explain your reasons if you disagree: (Optional)

1.5. I can easily imagine this system fitting naturally into my everyday activities and personal living space.

1	2	3	4	5
Strongly Disagree	Disagree	Neutral	Agree	Strongly Agree

Please explain your reasons if you disagree: (Optional)

1.6. I believe this system could be beneficial to my personal wellbeing.

1	2	3	4	5
Strongly Disagree	Disagree	Neutral	Agree	Strongly Agree

Please explain your reasons if you disagree: (Optional)

1.7. I think this system would be helpful for detecting feelings of loneliness and providing timely support.

1	2	3	4	5
Strongly Disagree	Disagree	Neutral	Agree	Strongly Agree

Please explain your reasons if you disagree: (Optional)

2. After experiencing the system and seeing the immediate data interpretation in the Deloneliness App, to which extent do you agree or disagree with the following statement:

2.1. I could easily understand the information shown in the Deloneliness App.

1	2	3	4	5
○	○	○	○	○
Strongly Disagree	Disagree	Neutral	Agree	Strongly Agree

If you disagree, please explain which part of the App you find difficult to understand?:

2.2. I was able to interpret the meaning of the displayed data without additional explanation.

1	2	3	4	5
○	○	○	○	○
Strongly Disagree	Disagree	Neutral	Agree	Strongly Agree

If you disagree, please explain which data you find difficult to understand?:

2.3. I trust that the health data measured by the system is accurate.

1	2	3	4	5
○	○	○	○	○
Strongly Disagree	Disagree	Neutral	Agree	Strongly Agree

Please explain your reasons if you disagree: (Optional)

2.4. I believe the system would reliably monitor my condition over time without frequent errors.

1	2	3	4	5
Strongly Disagree	Disagree	Neutral	Agree	Strongly Agree

Please explain your reasons if you disagree: (Optional)

2.5. I would feel comfortable sharing my data collected by this system with my family or healthcare team.

1	2	3	4	5
Strongly Disagree	Disagree	Neutral	Agree	Strongly Agree

Please explain your reasons if you disagree: (Optional)

2.6. I am comfortable with the idea of being continuously monitored by this system.

1	2	3	4	5
Strongly Disagree	Disagree	Neutral	Agree	Strongly Agree

Please explain your reasons if you disagree: (Optional)

3. Please rate the comfort level of the system components in the following:

How do you rate the comfort of the sensing garment?

Not at all comfortable	1	2	3	4	5	Very comfortable
	○	○	○	○	○	

How do you rate the comfort of the sensing bed sheet?

Not at all comfortable	1	2	3	4	5	Very comfortable
	○	○	○	○	○	

How do you rate the comfort of the sensing seat cushion?

Not at all comfortable	1	2	3	4	5	Very comfortable
	○	○	○	○	○	

Wearing or using the system felt supportive rather than restrictive.

Strongly Disagree	1	2	3	4	5	Strongly Agree
	○	○	○	○	○	

If you disagree, please specify which component(s) of the system you find uncomfortable?:

4. Overall Acceptance and Recommendation

4.1. Overall, I find this system acceptable to use.

1	2	3	4	5
○	○	○	○	○
Strongly Disagree	Disagree	Neutral	Agree	Strongly Agree

If you disagree, please specify which component(s) of the system you find unacceptable and requires improvement?:

4.2. I would recommend this system to my family, friends, or others.

1	2	3	4	5
○	○	○	○	○
Strongly Disagree	Disagree	Neutral	Agree	Strongly Agree

Please explain your reasons: (Optional)
