# Supplementary material for: Development and User-Centered Evaluation of Smart Systems for Loneliness Monitoring in Older Adults: Mixed Methods Study
Source: J Med Internet Res. 2026 Jan 28;28:e81027. doi: 10.2196/81027 (PMC12895156; doi:10.2196/81027)
Supplement: Multimedia Appendix 3 [file jmir_v28i1e81027_app3.pdf]

Name: Example

<table>
<tr><th>Time of Day</th><th>Daily Activities
Example</th><th>Emotions or Feelings
Example</th><th>Garment Improvement Ideas

Example</th><th>Furniture Improvement Ideas

Example</th></tr>
<tr><td>Morning

Example</td><td>1. Morning Exercise

2. Wear on Clothing</td><td>1. I need something light and breathable for morning exercise, and soft and warm at night.

2. My hands aren’t very nimble—I want something that’s easy to put on.</td><td rowspan="3">1. Choice of fabric, breathability, warmth, fit to body.
2. Fastening method: zipper, Velcro, magnetic closure, elastic band.
3. Acceptable sensor zones (chest, back, arms, etc.)/ Style and appearance (sportswear, loungewear, casual wear)
4. Other ideas?
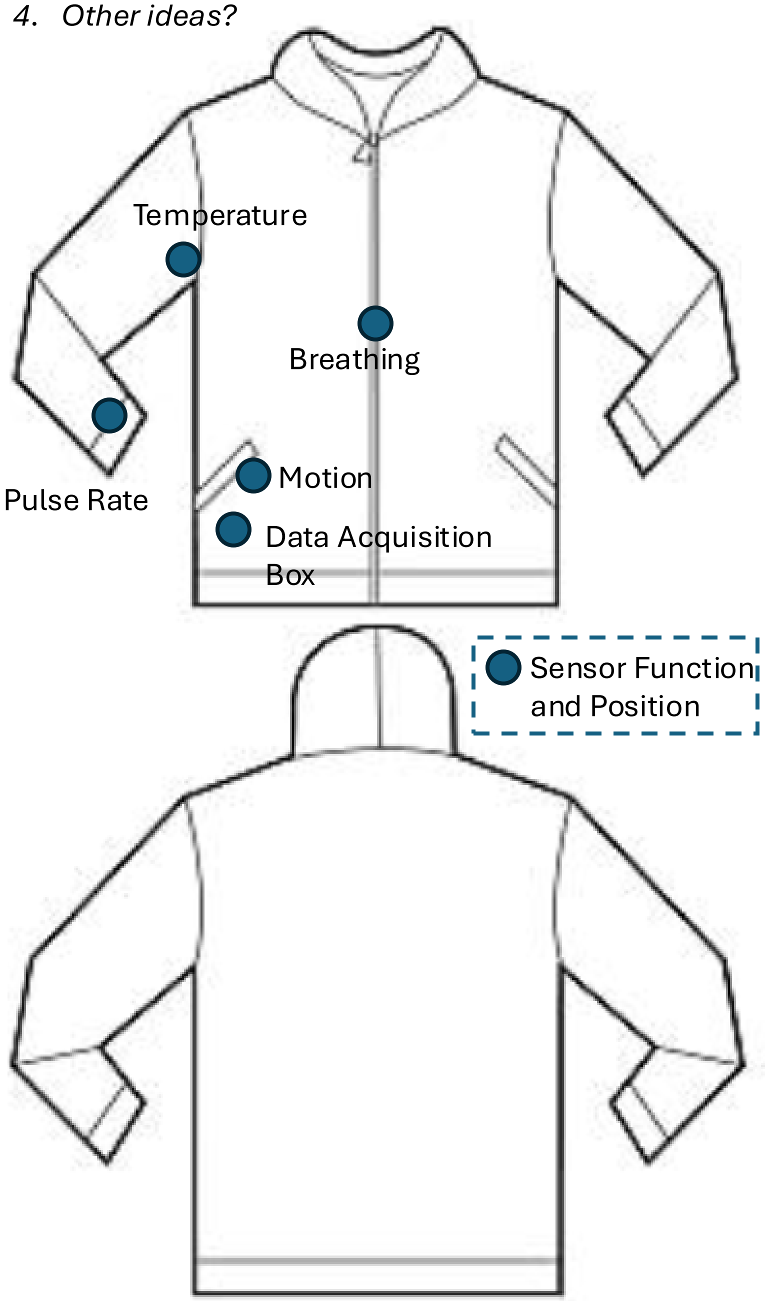
</td><td rowspan="3">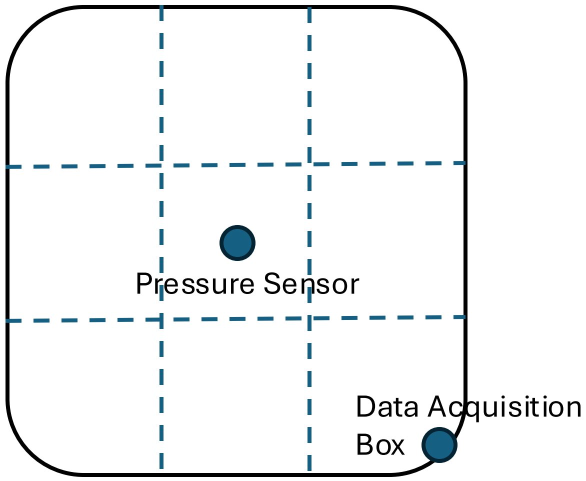

Sensing Seat Cushion

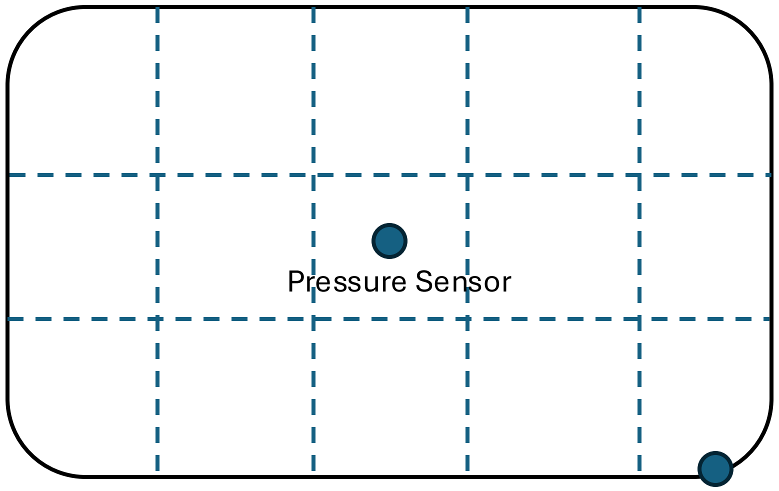

Data Acquisition Box
Sensing Pillow</td></tr>
<tr><td>Afternoon

Example</td><td>3. Take a Walk</td><td>3. I prefer not to have visible sensors on the front of my chest when I go outside wearing this garment./ I’d like it to look just like a normal workout top.</td></tr>
<tr><td>Evening

Example</td><td>4. Doing Laundry

5. Sleeping</td><td>4. I hope I don’t need to remove the battery every time I wash it.
5. I don’t feel comfortable wearing it to sleep—it feels like I’m being watched.</td></tr>
</table>

Name: ____________

<table>
<tr><th>Time of Day</th><th>Daily Activities</th><th>Emotions or Feelings</th><th>Garment Improvement Ideas</th><th>Furniture Improvement Ideas</th></tr>
<tr><td>Morning</td><td></td><td></td><td rowspan="3">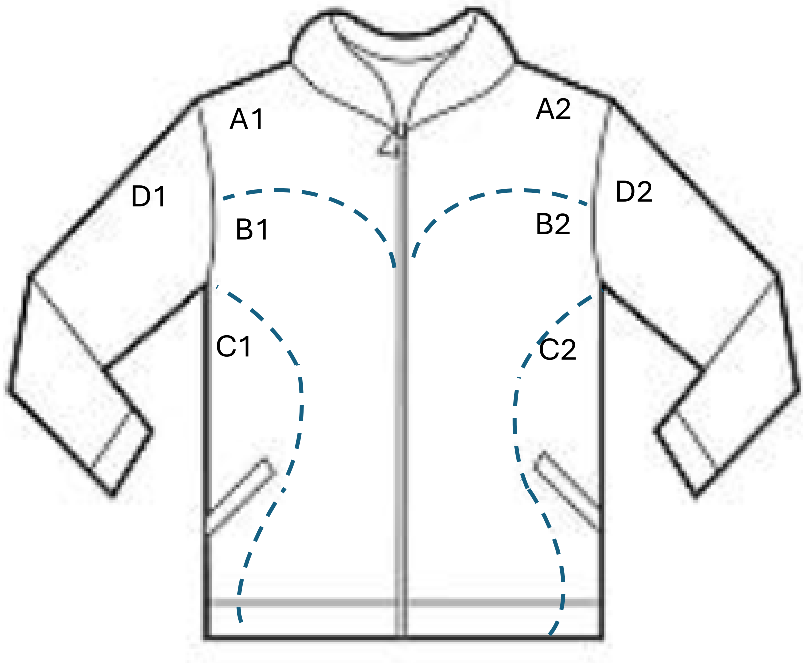

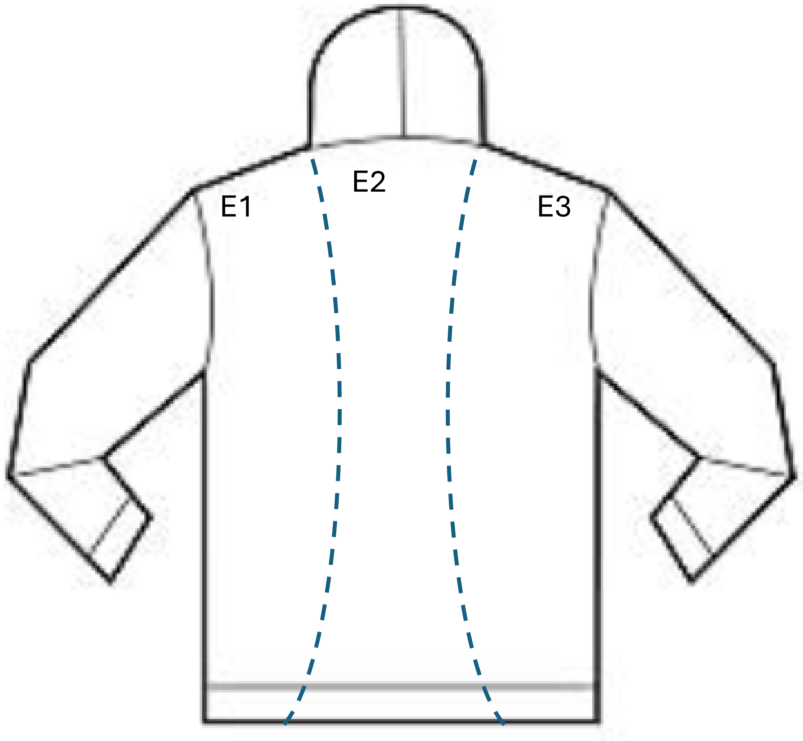
</td><td rowspan="3">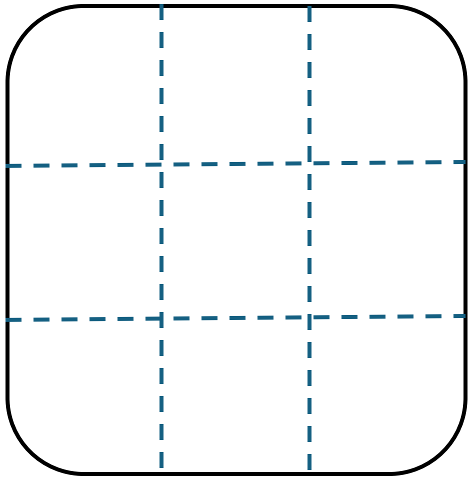
Sensing Seat Cushion
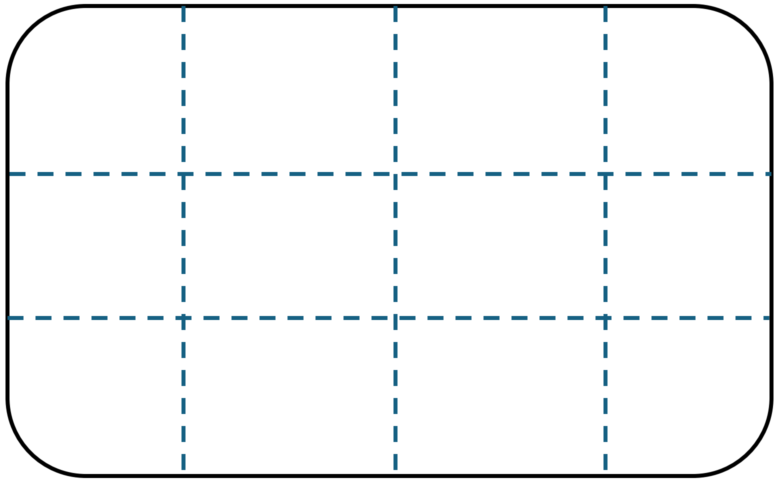
Sensing Pillow</td></tr>
<tr><td>Afternoon</td><td></td><td></td></tr>
<tr><td>Evening</td><td></td><td></td></tr>
</table>
